# Molecular Responses of Daphnids to Chronic Exposures to Pharmaceuticals

**DOI:** 10.3390/ijms24044100

**Published:** 2023-02-17

**Authors:** Katie O’Rourke, Beatrice Engelmann, Rolf Altenburger, Ulrike Rolle-Kampczyk, Konstantinos Grintzalis

**Affiliations:** 1 School of Biotechnology, Dublin City University, D09 Y5NO Dublin, Ireland; 2 Department of Molecular Systems Biology, Helmholtz-Centre for Environmental Research—UFZ, 04318 Leipzig, Germany; 3 Department of Bioanalytical Ecotoxicology, Helmholtz-Centre for Environmental Research—UFZ, 04318 Leipzig, Germany

**Keywords:** *Daphnia magna*, pharmaceuticals, chronic toxicity, metabolomics, glutathione-S-transferase, enzymes

## Abstract

Pharmaceutical compounds are among several classes of contaminants of emerging concern, such as pesticides, heavy metals and personal care products, all of which are a major concern for aquatic ecosystems. The hazards posed by the presence of pharmaceutical is one which affects both freshwater organisms and human health—via non-target effects and by the contamination of drinking water sources. The molecular and phenotypic alterations of five pharmaceuticals which are commonly present in the aquatic environment were explored in daphnids under chronic exposures. Markers of physiology such as enzyme activities were combined with metabolic perturbations to assess the impact of metformin, diclofenac, gabapentin, carbamazepine and gemfibrozil on daphnids. Enzyme activity of markers of physiology included phosphatases, lipase, peptidase, β-galactosidase, lactate dehydrogenase, glutathione-S-transferase and glutathione reductase activities. Furthermore, targeted LC-MS/MS analysis focusing on glycolysis, the pentose phosphate pathway and the TCA cycle intermediates was performed to assess metabolic alterations. Exposure to pharmaceuticals resulted in the changes in activity for several enzymes of metabolism and the detoxification enzyme glutathione-S-transferase. Metabolic perturbations on key pathways revealed distinct groups and metabolic fingerprints for the different exposures and their mixtures. Chronic exposure to pharmaceuticals at low concentrations revealed significant alterations of metabolic and physiological endpoints.

## 1. Introduction

The continuous global increase in population and consumption of resources has a significant impact to the environment due to anthropogenic activities. Attributed to an ageing population and prevalence of chronic illnesses, consumption of pharmaceuticals has continued to increase over the decades [1]. From 2000–2017 in OECD countries, the use of lipid regulators and anti-diabetic drugs alone tripled and doubled, respectively (OECD, 2019). As a result of this continued progression, combined with improvements in analytical techniques for pharmaceutical detection, pharmaceuticals have been widely detected in surface waters globally. Moreover, due to their pseudo-persistence and exposure in mixtures in the aquatic environment, pharmaceuticals are not required to be present in high concentrations to disrupt normal function of aquatic biota [2,3]. Pharmaceuticals commonly occur in the environment at small concentrations and complex mixtures and, in recent years, pharmaceuticals have been recognized as emerging contaminants of concern. In relation to water monitoring practices, over the last year these have shifted from quantification practices using analytical techniques such chromatography coupled with mass spectrometry towards effect-based methods, the basis of which involves measuring responses of sentinel species to a compound of interest (e.g., xenobiotic) or even a mixture of chemicals. Effect-based methods capture a particular status of a particular body of water and allow the timely prediction of pollution hot-spots before the damage becomes irreversible [4,5,6]. 

This change in practice has revealed the potential risk posed by xenobiotics to the normal function and development of non-target organisms. These enhanced practices are also referred to as New Approach Methodologies (NAMs), a term which encapsulates the use of “in chemico and in vitro assays, and in silico approaches”. When applied in combination with novel tools and analytical methods, NAMs can assist in chemical risk assessment and safety regulation [7]. The adoption of NAMs has certainly increased and is currently employed to inform decision making at EU and industry levels. Moreover, the movement towards non-animal testing approaches has increased the application of NAMs [8]. Among these approaches are omics technologies, for example metabolomics, proteomics and transcriptomics. Omics allow the identification of molecular signatures of surrogate species, phylogenetically related to humans, in response to complex chemical mixtures to replace traditional animal testing with evolutionarily diverse model organisms in the tree of life [9]. These unique signatures facilitate the identification of mechanisms of actions and targets of toxicity of chemicals via adverse outcome pathways [10], and informs about their hazards for higher organisms and humans. 

Pharmaceuticals find their way to the aquatic environment through several pathways, for example household and medical waste, where they may remain biologically active in the environmental space and threaten non-target organisms [11,12,13]. An obvious pathway of pharmaceutical pollution is via wastewater effluent, due to the inability of wastewater treatment plants to effectively remove small and relatively stable compounds. For this reason, e.g., carbamazepine and gemfibrozil are documented to have low removal rates and therefore are described as persistent [14]. Moreover, freshwater systems are often the receiving body for effluents from hospital and or industrial waste, while they serve also as the main source in drinking water production, leading to the entry of pharmaceuticals into domestic water supplies [15,16]. Other routes of contamination include surface run-off from agricultural or industrial land and improper disposal of drugs via the toilet [17,18,19]. Depending on physicochemical properties such as solubility, persistence and polarity, pharmaceuticals can eventuate into the aquatic ecosystem unchanged or as a transformation product, which in some cases can be more harmful than the original parent compound [20]. 

Among the aquatic organisms, crustaceans of the genus *Daphnia* have gained significant interest in ecotoxicology mainly due to their geographical distribution, the central role in food webs and adaptation to a range of habitats [21]. Daphnids can be easily cultured under laboratory conditions, which makes them ideal for laboratory experimentation. Additionally, daphnids were the first crustacean to have its genome sequenced, which was characterized as “ecoresponsive”, highlighting their ability to respond to environmental stimuli [22]. Up to now, there is a significant amount of genomic data and molecular knowledge accumulated on them, which makes daphnids ideal for experimentation [23]. Daphnids reproduce via a parthenogenic lifecycle in the lab and generate clonal female populations without genetic background differences. Therefore, they produce uniform responses to a toxin or stress among individuals, while being filter feeders they are extremely sensitive to changes in their aqueous environment, and hence suitable for detection exposures to xenobiotics. 

This study aimed to unveil the mechanisms of toxicity for several commonly detected pharmaceuticals and their mixture, specifically with chronic exposure at sub-lethal concentrations to simulate a realistic scenario. These pharmaceuticals represent a hazard for non-target organisms of the aquatic environment; however, biological data regarding their undesirable effects on aquatics fauna are lacking. To address this issue, and in an attempt to understand the mechanisms at play, daphnids were exposed to pharmaceuticals with different modes of action and the metabolic perturbations were assessed using targeted liquid chromatography coupled with mass spectrometry in combination with biochemical markers of their physiology. 

## 2. Results

**Figure 1:**
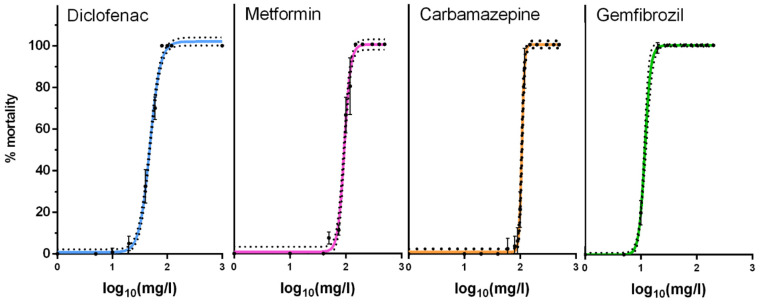
Toxicity curves of pharmaceuticals. Twenty neonates (<24 h) were exposed for 24 h to diclofenac, metformin, carbamazepine and gemfibrozil in 50 mL OECD media. Data represent average ± SD (N = 4) for each concentration.

Toxicity of pharmaceuticals was estimated in Daphnia neonates using concentrations response functions (Figure 1) from which the EC values were calculated (Table 1). Toxicity curves were generated for each pharmaceutical with the exception of gabapentin which did not induce any mortality even at extremely high concentrations (100 mg/L) and which would not be considered environmentally relevant for our system. Diclofenac proved to be the most toxic, prompting mortality at lower concentrations compared to its counterparts. However, overall, the pharmaceuticals demonstrated rather similar EC values when compared with each other (Table 1). 

To simulate the chronic exposures to pharmaceuticals, initially a concentration of 10 mg/L was tested; however, this concentration proved intolerable for daphnids with low survival after 7 days. Therefore, the chronic experiment was performed at 1 mg/L for exposures of individual pharmaceuticals or their combined mixtures. Furthermore, as only carbamazepine and gemfibrozil required DMSO as a carrier solvent, the chronic experiments were performed separately for DMSO (carbamazepine and gemfibrozil) and OECD media (diclofenac, metformin, gabapentin) dissolved pharmaceuticals and their mixtures. For biochemical assays and metabolomic analyses, daphnids following the 21 days exposure were collected and assayed for key enzyme activities for alkaline (ALP) and acid (ACP) phosphatases, β-galactosidase (BGAL), lipase (LIP), peptidase (PEP), lactate dehydrogenase (LDH) and glutathione-S-transferase (GST). The five pharmaceuticals were separated according to their solubility in the OECD medium; diclofenac, metformin, gabapentin and their mixture constituted one group which were freely soluble in OECD (Table 2), the other group was composed of carbamazepine, gemfibrozil and their mixture which required the carrier solvent DMSO (Table 3). 

ALP activity was impacted by diclofenac and metformin, both pharmaceuticals caused significant decreases of 15.2% and 18.1%, respectively. In addition, reductions in ACP activity were also recorded; diclofenac, gabapentin and the mixture decreased ACP activities by 27.3%, 13.6% and 18.2%. Metformin was the only pharmaceutical to affect βGAL activity, causing a decrease of 21.9%. Similarly, LIP, and PEP activities were only altered by a single pharmaceutical; gabapentin increased LIP activity by 21.3%, and diclofenac increased PEP activity by 24.1%. LDH activity was impacted by the pharmaceutical mixture alone, activity was significantly increased by 82.5%. GST was significantly increased by individual pharmaceuticals metformin (29.2%), gabapentin (31.2%) and by the pharmaceutical mixture (41.4%). 

ALP and LIP activity was significantly altered by the carrier solvent DMSO alone, increasing activity in both cases by 21.6% and 20.6%, respectively. ACP was also increased by DMSO (10.3%); however, carbamazepine and gemfibrozil induced opposite effects, causing decreases of 15.6% and 9.4%. Gemfibrozil was responsible for the only significant differences in BGAL activity recorded, specifically a 38.9% increase. Peptidase activity was significantly altered by each pharmaceutical, the mixture and carrier solvent. DMSO caused a 73.7% increase whereas carbamazepine, gemfibrozil and the mixture caused decreases of 45.8%, 28.9% and 31.6%, respectively. Changes in the activity of LDH were recorded for DMSO and the mixture, DMSO increased activity by 89% whereas the pharmaceutical mixture caused a 23.6% decrease. Similarly, to peptidase, GST was significantly decreased by carbamazepine (43%), gemfibrozil (10.3%) and the mixture (15.6%), whereas DMSO increased GST activity by 41.4%. 

**Figure 2:**
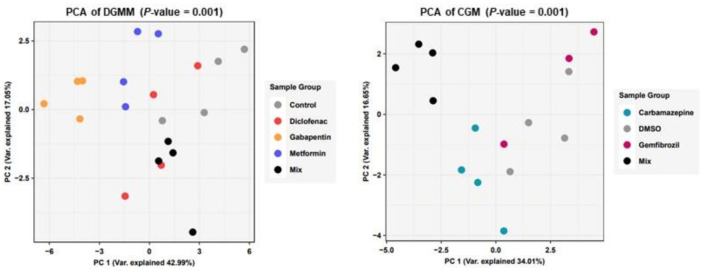
Principal component analysis of metabolite quantifications for different exposure groups of pharmaceuticals. PCA using metabolite abundances as input was performed in R. Differences between treatment groups were calculated with Permanova using Adonis function.

These observations in relation to the physiology demonstrated responses of daphnids with enhanced levels of metabolic perturbations regarding the central carbon metabolism focusing on glycolysis, pentose phosphate pathway and the TCA cycle. Specifically, a targeted approach for the analysis of key metabolites from these pathways revealed significant metabolic differences employing multivariate analysis of their fingerprints (Figure 2). Multivariate statistical analysis revealed distinct groups among the different exposures. For the dataset of aqueous dissolved pharmaceuticals, OECD controls separate on the principal component 1 (PC1) axis from metformin and even further from gabapentin. Moreover, a separation on PC2 is observed for the mixture of all the aforementioned pharmaceuticals. Regarding the second dataset, for carbamazepine and gemfibrozil, there is a clear separation of the mixture on PC1 axis from all groups, while DMSO seems to have an intermediate effect. 

**Figure 3:**
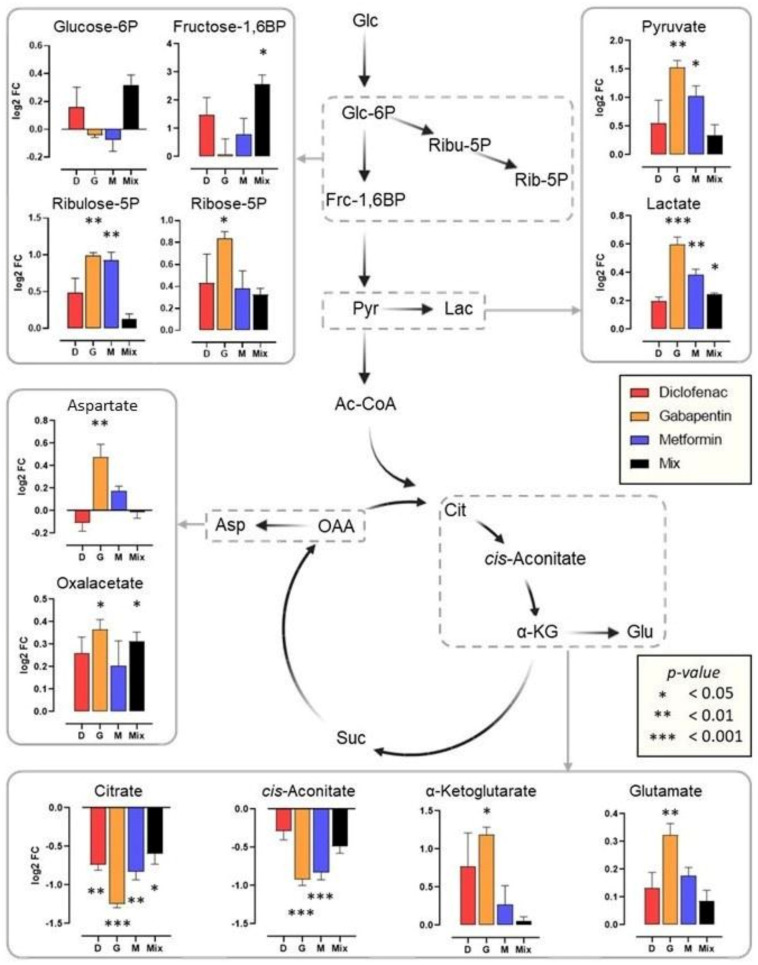

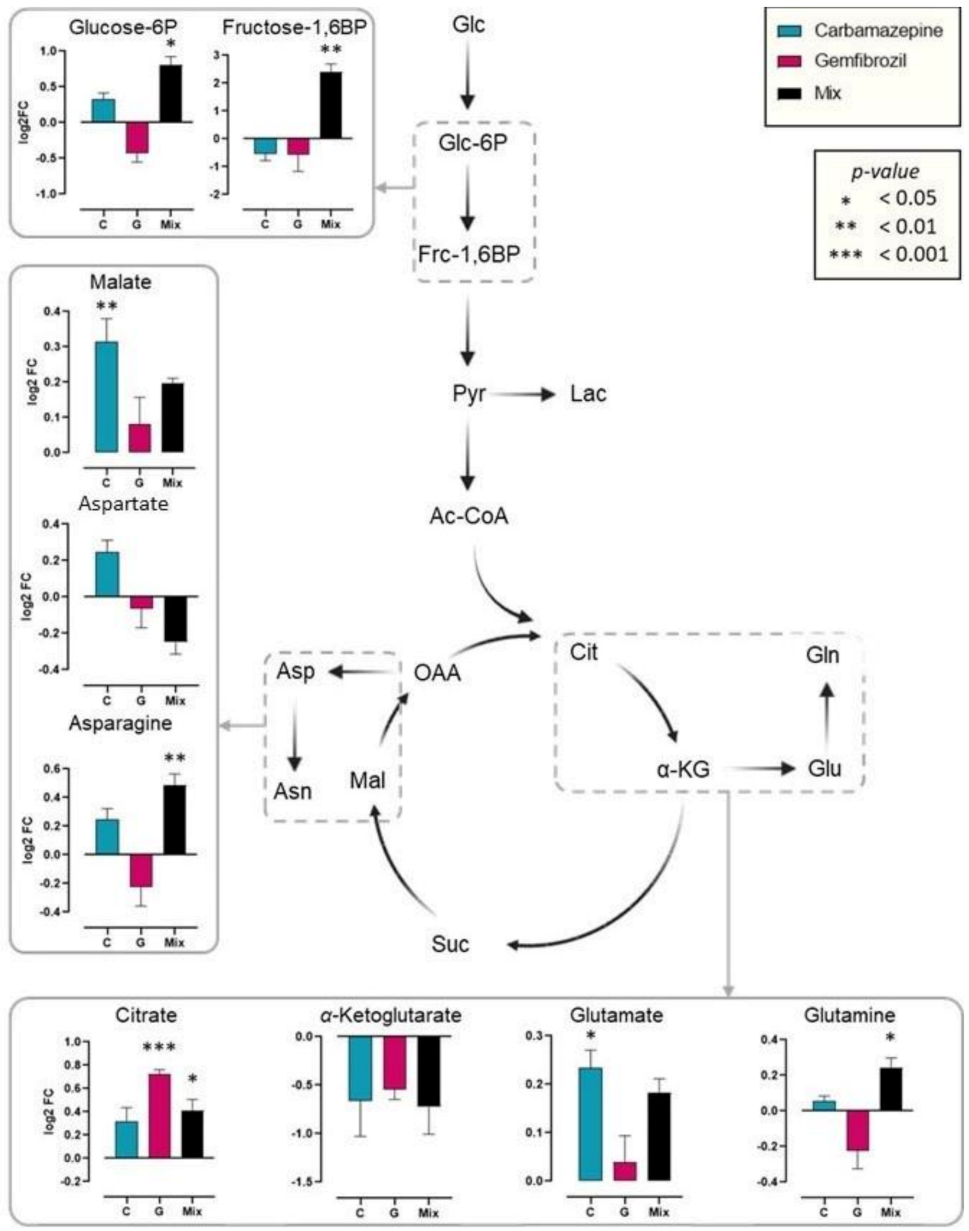
Illustrating the metabolic impact of pharmaceuticals on daphnids. A. Statistical significance is displayed for diclofenac, gabapentin, metformin and their mixture compared with the OECD control. B. Carbamazepine, gemfibrozil and their mixture are compared with DMSO control used as their carrier solvent. Glc = glucose, Glc-6P = glucose 6-phosphate, Ribu-5P = ribulose 5-phosphate, Rib-5P = ribose 5-phosphate, Frc-1,6BP = fructose 1, 6-bisphosphate, Pyr = pyruvate, Lac = lactate, Ac-CoA = acetyl coenzyme A, Cit = citrate, α-KG = α-ketoglutarate, Glu = glutamate, Gln = glutamine, Suc = succinate, Mal = malate, OAA = oxaloacetate, Asp = aspartate, Asn = asparagine. Statistical significance was calculated by ANOVA followed by Tukey’s test for multiple comparisons. * *p* < 0.05, ** *p* < 0.01, *** *p* < 0.001. The log2FC values used in the metabolic network reconstruction figure are provided in the Supplementary Materials.

A reconstruction of the central metabolic pathways indicated above revealed alterations in metabolite abundances of the glycolysis, the pentose phosphate pathway and the TCA cycle (Figure 3). Exposure to aqueous pharmaceuticals resulted in decreased levels of citrate and cis-aconitate, which is accompanied by an increase in α-ketoglutarate, subsequently indicating a flux towards glutamate. In addition, there is an increase towards oxaloacetate; however, only for gemfibrozil and metformin. This was accompanied with a shift towards aspartate; therefore, a diversion from the TCA cycle. For glycolysis, glucose-6-phosphate was increased under the diclofenac and the mixture exposure but decreased in gabapentin and metformin experiments, however, in all exposures a shift towards the pentose phosphate pathway was observed as deduced from the increase for ribulose-5-phosphate and ribose-5-phosphate. Chronic exposure to all DMSO soluble pharmaceutical resulted in an increase in citrate, malate and glutamate and a decrease in α-ketoglutarate. However, for other metabolites there were more treatment-specific responses. Specifically, asparagine and glucose-6-phosphate were decreased for gabapentin exposure, but this was reversed for carbamazepine and the mixture. 

## 3. Discussion

Freshwater organisms are non-target species of the aquatic ecosystem that are potentially adversely impacted by pharmaceutical pollutants.

Diclofenac is a potent NSAID, that is widely used in oral and topical forms (i.e., ointments) which find numerous applications in both human and veterinary medicine. As one of the most commonly detected pharmaceuticals in environmental matrices, diclofenac has been extensively researched and has been documented to interfere with the physiology of non-target organisms and particularly invertebrates [24,25,26,27]. Significant changes related to biochemical and phenotypic endpoints have been reported such as decrease in the enzyme activity of cholinesterase and selenium dependant glutathione peroxidase in daphnids [28], although in other studies opposite effects for increases of glutathione peroxidase and lipid peroxidation and a decrease of superoxide dismutase (SOD) and DNA damage were reported after 48 and 96 h exposure [29]. In general, oxidative damage through the overproduction of ROS increased acetylcholinesterase activity and decreased ingestion and filtration rates have also been reported [30], as well as changes in expression of genes related to metabolism, growth, development and reproduction [31]. These responses of course are dependent on the concentration and type of exposure. For example, exposure to diclofenac for longer periods has shown to affect the timing of first egg production and time to first brood [31]. In our study, diclofenac significantly affected enzymes related to the central energy metabolism, and specifically ALP, ACP and PEP and decreased metabolites such as citrate and aconitate, while increasing α-keto glutarate and glutamate, indicating a metabolic shift away from the TCA cycle. In a recent study on the aquatic invertebrate *Hyalella azteca*, a metabolomic analysis showed the expected inhibition of prostaglandin synthesis but also an impact on the carnitine shuttle pathway and a similar mechanism of action to humans [32]. Furthermore, it has been reported that diclofenac can also affect plant metabolism, in particular the methylerythritol phosphate pathway of plastids, as well as decrease stomatal conductance and net assimilation rate as diclofenac concentration increased [33]. In light of this, what should also be taken into account in the study of such drugs is their biotransformation as this is directly linked with their toxicity induced effects. Especially for diclofenac, the generation of taurine conjugate and methyl ester has been validated in invertebrates and showed significant differences in toxicity of these compounds [34]. 

Metformin, is derived from galegine, a natural product isolated from the medicinal plant *Galega officinalis* [35]. The common name of metformin in the market is Glucophage, and is a pharmaceutical medication administered as the first line of treatment in diabetes type 2 patients. Attributed to the global diabetes epidemic, the consumption of metformin is continuously increasing. For this reason, metformin has become ubiquitous in environmental matrices and is frequently detected in the aquatic ecosystem at concentrations two orders of magnitude higher than other pharmaceuticals [36]. Although metformin is regarded as an environmentally relevant pharmaceutical, there are limited studies reporting its effect on non-target organisms; however, the minimal literature available has revealed the impact of metformin on aquatic invertebrates and fish [37,38]. At low concentrations, metformin was responsible for phenotypic alterations of daphnids including a decrease in lifespan, while at low concentration the lifespan was increased as it was also the case in some other species [39]. Although, structurally different to hormones, specifically oestrogens, metformin is considered to elucidate effects similar to an endocrine disrupting compound (EDC) [40]. Studies have revealed its potential as an EDC, whereby male fathead minnow displayed intersex characteristics such as oocytes presenting in the testes, and the fish pairs with an intersex male produced fewer and smaller clutches [41]. Similarly, in Japanese rice fish, metformin induced the occurrence of intersex females as well as impacting the biochemical system by increasing the production of reactive oxygen species, decreasing glutathione (male fish) and increasing catalase (CAT) activity (female fish) [40]. Taking into account that the main pharmacological action of metformin is exerted via the activation of adenosine monophosphate kinase (AMPK), a suppressor of the electron transport chain complex I in mitochondria, an interference with cellular energy balance would be expected. In this study, metformin impacted several enzymes, substantially increasing ALP and GST activity and significantly decreased βGAL activity. The assessment of the metabolic perturbations revealed that several metabolites were also impacted by metformin and specifically, in the glycolytic pathway, ribulose-5-phophate was increased as well as pyruvate and lactate. Citrate and cis-aconitate on the other hand were significantly decreased such as in the case of diclofenac, indicating a shift to increased energy use. These findings are in agreement with a metabolomic study in mouse embryonic fibroblast cells, a metabolic reprogramming was observed by the suppression of the TCA cycle and the elevated production of lactate and a decrease in the ration of NAD^+^/NADH [42]. 

Gabapentin is a synthetic amino acid used as anticonvulsant to treat seizures and neuropathic pain of individuals with epilepsy. Alike many other pharmaceutical drugs, the consumption rate of gabapentin is continuously increasing. In conjunction with its slow metabolism in humans and its poor removal rate from wastewater treatment plants (WWTPs), gabapentin has become an emerging contaminant of concern for freshwater ecosystems. There is scarce literature surrounding the non-target effects of gabapentin on hydrobionts and in particular on daphnids. However, experimental studies have revealed the toxicological effects gabapentin elucidates in zebrafish embryos with phenotypic endpoints such as swimming behaviour, body length and heart rate being significantly impacted upon exposure to the anticonvulsant, as well as the development of malformations of organs. Moreover, at environmentally relevant concentrations the enzymes CAT, LDH and GST were significantly altered [43]. Furthermore, in a transcriptomics-based analysis using zebrafish embryos, revealed changes in the expression of 130 and 750 genes related to antioxidant, immune and nervous systems [44]. Our study showed that upon chronic exposure to gabapentin, the enzymes ALP, LIP and GST were most sensitive. On a metabolic level, gabapentin proved to be the pharmaceutical with the largest impact on almost every metabolite detected with the exception of glucose-6-phosphate and fructose-1,6-biphosphate in the glycolysis pathway thus indicating a strong disruption of the central carbon metabolism upon exposure. 

The anticonvulsant carbamazepine is a commonly detected pharmaceutical in WWTPs influent, effluent and in some instances even in drinking water. As a result, it is considered ubiquitous and has been discovered up to the µg/L range in surface waters and in some regions had a 100% detection rate in rivers [45]. The literature reports the ability of carbamazepine to inflict phenotypic and biochemical alterations in aquatic biota including daphnids. A chronic study in *Daphnia magna* reported reduced fecundity, fertility ad growth rate of daphnids upon exposure [46]. Similarly in another chronic study, a decline of reproductive output, moulting frequency and the production of male offspring was recorded [47]. However, the opposite effect was found in a study employing *Daphnia pulex*, where daphnids exposed to carbamazepine (1 µg/L) matured earlier and at certain body lengths produced more offspring when compared to the control, yet at higher concentrations (100 and 200 µg/L) population growth also decreased by 9% and 32% when compared to the control and daphnids exposed to the lower levels of the pharmaceutical (<10 µg/L) [48]. Other phenotypic endpoints reportedly impacted by carbamazepine include feeding behaviour such as ingestion and filtration rates and phototactic behaviour [30]. In the same study, several enzyme markers were assessed and exposure to carbamazepine resulted in significant reductions of SOD, AchE, CAT, and GST enzyme activities. Similarly, in this study, GST activities were reduced as was ACP and PEP activity. In addition, the analysis of central metabolic pathways revealed increased levels of malate and glutamate indicating only minor and very specific impacts on the carbon metabolism. There has yet been no metabolomic study in daphnids, however, in a recent study on mussels combining metabolomics and proteomics, low and high exposure concentration of carbamazepine resulted in consistent metabolic and protein signatures at both concentrations [49]. These findings strengthen the use of aquatic species and holistic approaches for the mechanistic understanding and prediction of biological responses to pharmaceutical occurrence in the environment. 

Gemfibrozil is a ligand of peroxisome proliferator-activated receptor α (PPARα) and acts as a fibrate drug for dyslipidemia in humans. The high prevalence of obesity in society has caused a steady incline of the consumption of gemfibrozil, and similar to the other pharmaceuticals of this study, there is limited information regarding the impact of gemfibrozil on non-target organisms; however, there is evidence of its toxicity to vertebrate and invertebrate organisms [50]. Exposure to the fibrate at low concentrations (50 ng/L) resulted in changes in phenotypic endpoints and life history parameters including increased mass, length and neonate production in daphnids, and at higher concentrations (500 ng/L) an increase in cholesterol levels was also detected [51]. However, in another study, exposing daphnids to gemfibrozil between 0.1–7.5 mg/L at varying temperatures observed the opposite affects. As temperature increased, reproductive outputs decreased and at 7.5 mg/L cholesterol levels decreased [52]. The biochemical impact of gemfibrozil in daphnids is not well reported; however, a study using the mussel *Dreissena polymorpha* showed the ability of gemfibrozil to cause biochemical alterations. Exposure to gemfibrozil within the µg/L range resulted in increased GST and metallothionein activities, increased lipid peroxidation and DNA damage after 96 h [53]. In contrast, our study revealed that chronic exposure of daphnids to gemfibrozil caused decreases in GST, ACP and PEP activity, and led to an increase in the activity of BGAL. Moreover, the metabolic assessment showed that prolonged gemfibrozil exposure resulted in increased citrate levels. There are no available data on metabolic perturbations of gemfibrozil to daphnids; however, in mice, gemfibrozil increased bile acid formation which was also connected with the observed liver toxicity [54]. 

The concept of the “cocktail” or mixture effect assesses the joint impact of a number of chemicals on a test species, which as an approach simulates more realistically the environment, where pharmaceuticals are undeniably found in combination with other chemicals. Thus, studying mixtures aims at providing a greater insight to the toxicity potentials of pharmaceutical cocktails. In this study, the triple mixture containing diclofenac, gabapentin and metformin did not clearly induce a greater impact to the enzyme markers or changes to the metabolites assessed; however, with some markers the mixture generated the greatest increase or decrease in an enzyme’s activity, as for example for GST and LDH. This is in agreement with [55], who observed that a pharmaceutical mixture did not cause more potent effects to daphnids in comparison to the individual compounds. However, it should be noted that the contrary has been observed in other pharmaceutical mixture studies [56,57]. For the DMSO soluble chemicals, the double mixture was responsible for decreases in PEP, LDH and the detoxification enzyme GST. Compared to the individual DMSO soluble compounds, the binary mixture induced the most significant changes within the metabolome of the daphnids, including increased fructose-1,6BP, asparagine, citrate and glutamine. This was a major difference, as the mixture had rather smaller effects in the aqueous dissolved chemicals whereas the mix of DMSO dissolved pharmaceuticals seems to provoke an additive response. No matter the case, a strong effect on F16BP on both data sets indicates a junction between glycolysis and the pentose phosphate pathway and a disruption in metabolic flow. What is also notable is that citrate demonstrated reversed effects in the two types of datasets and there were more profound effects on the aqueous dissolved chemicals. 

Metabolomics, along with other holistic omic techniques are now accepted as valuable tools for water monitoring. Metabolomics specifically is useful in an ecotoxicology scenario, as an organism’s metabolism is their first line of defines to xenobiotics [58,59]. We recently highlighted the use of metabolic signatures in daphnids combined with physiologic markers as endpoints used in toxicity of mixtures of pollutants [60]. Our study employed a combination of physiological endpoints and a metabolomic analysis to identify targets of toxicity for several pharmaceuticals at a molecular level. However, our study only assessed several key enzymes of metabolism and one of detoxification (GST), further assessment could include additional detoxification enzymes such as catalase, glutathione reductase and peroxidase and superoxide dismutase to complete the picture in response to oxidative stress. Furthermore, as a pilot study, only the central metabolic pathways (glycolysis, TCA and pentose phosphate pathway) were covered, and potentially a future more expansive metabolome assessment could provide greater insight to the whole toxicity potential of these compounds. 

## 4. Materials and Methods

### 4.1. Reagents

All chemicals used in this study were of the highest purity. 

Erythromycin, 1,1dimethylbiguanide hydrochloride (metformin), gemfibrozil, *p*-nitrophenyl phosphate, disodium salt, hexahydrate (pNPP), and calcium chloride dihydrate were purchased from Thermo Scientific. Trimethoprim, diclofenac sodium, gabapentin, and carbamazepine were purchased from Acros Organics. Amoxicillin, albumin from bovine serum (BSA), L-glutathione reduced (GSH), 1-chloro,24-dinitrobenzene (CDNB), potassium chloride, 2-nitrophenyl-β-D-galactopyranoside (ONPG), *p*-nitrophenyl butyrate (pNPB) were purchased from Sigma Aldrich. L-Leucine-4-nittoanilide, beta-nicotinamide adenine dinucleotide reduced (NADH) were purchased from Alfa Aesar. Sodium pyruvate, magnesium sulfate heptahydrate, and sodium hydrogen carbonate were purchased from Fisher Scientific. 

### 4.2. Culturing Daphnids and Exposures to Pharmaceuticals

Daphnids were cultured in conformity with OECD guidelines in 4 L beakers in OECD media (final concentrations 0.29 g CaCl_2_.2H_2_O/l, 0.123 g MgSO_4_.7H_2_O/l, 0.065 g NaHCO_3_/l, 0.0058 g KCl/l, 2 μg Na_2_SeO_3_/l, pH 7.7) [60] under a 16h:8h of light:dark photoperiod at 21 °C. Breeding cultures of daphnnids were fed with an algal suspension (*Chlamydomonas rheinhartii*) and supplemented with dried baker’s yeast and an organic seaweed extract (*Ascophylum nodossum*) upon media renewal every four days. For acute toxicity exposures, twenty neonates (<24 h) from the third brood were exposed to each pharmaceutical in 50 mL OECD for 24 h and mortality (as immobilization) was recorded. Toxicity curves were plotted, and EC values were calculated. All plots were calculated based using the Four parameter logistic (4PL) model, following the equation Span = Top − Bottom and Y = Bottom + (Top-Bottom)/(1 + 10^((LogIC50-X)*HillSlope)), using the GraphPad software. The parameters top and bottom were commonly fixed to 100 and 0, accordingly. 

Having defined the toxicity potential for each pharmaceutical, for chronic exposures, twenty-four neonates (<24 h) were cultured until 21 days old in exposure vessels of 900 mL for single and mixture of chemicals at 1 mg/L. For aqueous soluble pharmaceuticals (diclofenac, gabapentin, metformin) OECD was the control, whereas for DMSO soluble pharmaceuticals (carbamazepine and gemfibrozil), OECD was the unexposed control and DMSO was tested as the carrier solvent at 0.0055%. All cultures were fed daily with algae and media was renewed every three days. 

**Figure 4:**
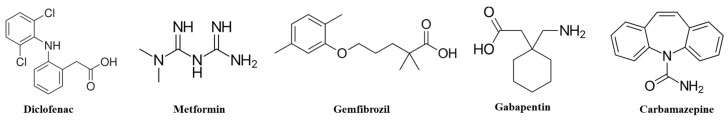
Pharmaceutical compounds used in this study.

The pharmaceutical compounds (Figure 4) diclofenac (non-steroidal anti-inflammatory drug), metformin (anti-diabetic), gemfibrozil (lipid-regulator), gabapentin and carbamazepine (anti-convulsant) were selected for their known different specific mechanisms of action in target organisms and their relevance as emerging contaminants of concern. 

### 4.3. Sample Homogenization for Biochemical Assays

From each exposure (or control) condition, four individuals were pooled together (to average any biological variation and increase sample) and stored for analysis in liquid nitrogen. Samples were homogenized in 0.5 mL ice-cold buffer depending on the enzyme assay using an Eppendorf pestle homogenizer, and cleared with centrifugation at 20,000× *g* at 4 °C for 10 min. The clear supernatant was split in aliquots and assayed immediately for enzyme activities as follows. Alkaline and acid phosphatases were quantified by the production of *p*-nitrophenol using *p*-nitrophenyl phosphate (pNPP) as a substrate at pH 9.8 or 4.5, respectively. β-galactosidase activity was assessed from the concentration of *o*-nitrophenol released from *o*-nitrophenyl-β-galactosidase (ONPG) at pH 7.2 phosphate buffer [61]. Lipase activity was assessed by the release of *p*-nitrophenol from *p*-nitrophenyl-butyrate at pH 7.2 phosphate buffer. Peptidase activity was monitored by the release of 4-nitroaniline from the hydrolysis of L-Leu-4-nitroanilide in pH 7.2 phosphate buffer. Lactase dehydrogenase activity was assessed from the decomposition of NADH in monitoring the kinetics of the reaction of pyruvate towards lactate at 340 nm [61]. Glutathione-S-transferase was quantified from the conjugation of GSH to 1-chloro-2,4-dinitrobenzene monitored photometrically at 340 nm [62,63]. Enzyme activity was expressed as enzyme units per protein quantified by a sensitive Bradford assay [64]. Statistically significant differences between exposures and unexposed (OECD) control and the carrier solvent were assessed following a Student’s *t*-test. 

### 4.4. Sample Homogenization for Metabolomics Analysis

Following exposures, three individuals were frozen immediately for analysis in liquid nitrogen. Samples were homogenized in 0.6 mL ice-cold methanol: water (4:1; both HPLC-grade) using an Eppendorf pestle homogenizer to quench metabolic reactions. The homogenates were cleared by centrifugation at 10,000× *g* at 4 °C for 5 min and the 100 µL of the clear supernatant was vacuum dried using a speedvac and stored at −80 °C until further analysis. Targeted LC-MS/MS data of each extract was acquired on a QTRAP 6500+^®^ system (Sciex, Framingham, MA, USA) coupled online with an HPLC-system. First, each sample was resuspended in 120 µL water and 10 µL were injected onto an Agilent 1290 II infinity UPLC system (Agilent Technologies Inc., Santa Clara, CA, USA). Chromatographic separation was achieved using a XSelect HSS T3 XP column (2.1 × 150 mm, 2.5 µm, 100 Å; Waters, Milford, MA, USA). Metabolites were eluted at a flow rate ranging from 0.4 mL/min to 0.15 mL/min with a non-linear 33 min gradient. Mobile phase A and B were 10 mM tributylamine, 10 mM acetic acid, 5% methanol and 2% 2-propanol (pH 7.1) and 100% 2-propanol, respectively. Autosampler was kept at 5°C and column oven was set at 40 °C. Identification and relative quantification were based on specific MRM transitions measured in negative mode electrospray ionization. Data acquisition and analysis was performed in Analyst^®^ software (Version 1.7.1). All further analysis were performed in in-house written R scripts. 

## 5. Conclusions

Recently, we highlighted the role of daphnids as key species in molecular ecotoxicology and the application of metabolomics in pollution monitoring as a sensitive endpoint [60]. This study revealed distinct changes in the polar metabolic profile of daphnids exposed to various pharmaceuticals at sub-lethal concentrations, as well as significant changes in multiple biochemical markers. In particular, the anticonvulsant, gabapentin was responsible for the most differences in metabolite levels, these changes were coupled with alterations in several enzyme activities. The employment of the sentinel species *Daphnia magna* in ecotoxicology studies offers an alternative to animal testing in the risk assessment of a chemical, and in this approach daphnids act as the “canary in the coal mine” within aquatic environments [65] providing profound insight to the toxicity potential of chemicals. We envisage that when applied in real-life settings, these novel approaches for monitoring will allow the timely prediction of pollution in the aquatic environment before it reaches irreversible levels. 

## Figures and Tables

**Table 1:** Effect concentration in mg/L for diclofenac, metformin, carbamazepine and gemfibrozil, calculated for neonates exposed to pharmaceuticals for 24 h.* The Hill model used is provided in Materials and Methods.

Chemical	Hill Slope	EC_50_	EC_10_	EC_5_	EC_1_
Diclofenac	4.6	101.3	62.8	53.4	37.3
Metformin	7.76	99.04	74.6	67.8	54.8
Carbamazepine	18.21	107.5	95.3	91.5	83.5
Gemfibrozil	8.3	100	76.7	70.1	57.5

* presented precision does not signal significance but serves the purpose for reusability. EC = Effect concentration.

**Table 2:** The chronic impact of diclofenac, metformin, gabapentin and their mixture on key enzyme activities of daphnids.

Enzyme	Control	Diclofenac	Metformin	Gabapentin	Mixture
ALP	13.8 ± 1.42	11.7 ± 0.82 (−15.2%) *	11.3 ± 0.71 (−18.1%) *	13.4 ± 1.47	11.8 ± 1.66
ACP	4.4 ± 0.34	3.2 ± 0.01 (−27.3%) *	4.1 ± 0.41	3.8 ± 0.24 (−13.6%) *	3.6 ± 0.28 (−18.2%) *
βGAL	3.2 ± 0.36	2.7 ± 0.32	2.5 ± 0.29 (−21.9%) *	2.8 ± 0.14	3.1 ± 0.26
LIP	12.2 ± 0.44	12 ± 1.43	14.1 ± 2.4	14.8 ± 0.38 (+21.3%) *	12 ± 1.45
PEP	137 ± 14.4	170 ± 11.3 (+24.1%) *	148 ± 10.3	143 ± 15.6	154 ± 13.5
LDH	39.4 ± 8.36	31.2 ± 6	50.1 ± 4.23	46.2 ± 6.71	71.9 ± 11.2 (+82.5%) *
GST	39.4 ± 2.57	34.5 ± 11	50.9 ± 2.12 (+29.2%) *	52 ± 4.62 (+31.2%) *	55.6 ± 1.72 (+41.1%) *

Enzyme activity was expressed as units/mg protein for alkaline and acid phosphatases, β-galactosidase and lipase, and as munits/mg protein for peptidase, lactate dehydrogenase and glutathione-S-transferase. ALP = alkaline phosphatase, ACP = acid phosphatase, β-GAL = β-galactosidase, LIP = lipase, PEP = peptidase, LDH = lactate dehydrogenase, GST = glutathione-S-transferase. Data represents average ± SD (N = 4) replicates for each condition. The asterisk (*) indicates a statistically significant difference by Student’s *t*-test compared to the unexposed control and the numbers in parenthesis indicate the percent difference.

**Table 3:** The chronic impact of carbamazepine, gemfibrozil and their mixture on key enzyme activities of daphnids.

Enzyme	Control	DMSO	Carbamazpine	Gemfibrozil	Mixture
ALP	3.7 ± 0.33	4.5 ± 0.42 $	3.9 ± 0.31	5 ± 0.18	3.9 ± 0.28
ACP	2.9 ± 0.13	3.2 ± 0.19 $	2.7 ± 0.2 (−15.6%) *	2.9 ± 0.02 (−9.4%) *	3 ± 0.11
βGAL	1.6 ± 0.23	1.8 ± 0.19	1.9 ± 0.2	2.5 ± 0.22 (+38.9%) *	2 ± 0.12
LIP	71.5 ± 6.71	86.2 ± 4.7 $	84.4 ± 6.35	82.4 ± 3.56	92.2 ± 6.15
PEP	319 ± 55.4	554 ± 15.5 $	300 ± 78.1 (−45.8%) *	394 ± 33.2 (−28.9%) *	379 ± 16.6 (−31.6%) *
LDH	78.3 ± 13.3	148 ± 23.4 $	154 ± 32.5	126 ± 14.9	113 ± 10.9 (−23.6%) *
GST	56.3 ± 10.8	79.6 ± 6.07 $	45.4 ± 6.88 (−43%) *	71.4 ± 0.74 (−10.3%) *	67.2 ± 7.83 (−15.6%) *

Enzyme activity was expressed as units/mg protein for alkaline and acid phosphatases, β-galactosidase and lipase, and as munits/mg protein for peptidase, lactate dehydrogenase and glutathione-S-transferase. ALP = alkaline phosphatase, ACP = acid phosphatase, β-GAL = β-galactosidase, LIP = lipase, PEP = peptidase, LDH = lactate dehydrogenase, GST = glutathione-S-transferase. Data represents average ± SD (N = 4) replicates for each condition. The asterisk (*) indicates statistically significant difference by Student’s *t*-test compared to DMSO and the numbers in parenthesis indicate the percent difference. For DMSO, a comparison with the unexposed control was designated by $.

## Data Availability

Not applicable.

## Supplementary Materials

The following supporting information can be downloaded at: https://www.mdpi.com/article/10.3390/ijms24044100/s1.
